# Estimation of Occupational Exposure to Asbestos in Italy by the Linkage of Mesothelioma Registry (ReNaM) and National Insurance Archives. Methodology and Results

**DOI:** 10.3390/ijerph17031020

**Published:** 2020-02-06

**Authors:** Chiara Airoldi, Daniela Ferrante, Lucia Miligi, Sara Piro, Giorgia Stoppa, Enrica Migliore, Elisabetta Chellini, Antonio Romanelli, Carlo Sciacchitano, Carolina Mensi, Domenica Cavone, Elisa Romeo, Stefania Massari, Alessandro Marinaccio, Corrado Magnani

**Affiliations:** 1 Unit of Medical Statistics and Cancer Epidemiology, Department of Translational Medicine, University of Eastern Piedmont, Novara, CPO-Piedmont, 28100 Novara, Italy; chiara.airoldi@uniupo.it (C.A.); daniela.ferrante@uniupo.it (D.F.); 2 Tuscany Regional Operating Centre of Low Etiological Fraction Occupational Cancer, Occupational and Environmental Epidemiology Branch, Cancer Risk Factors and Lifestyle Epidemiology Unit, Institute for Cancer Research, Prevention and Clinical Network (ISPRO), 50141 Florence, Italy; l.miligi@ispro.toscana.it (L.M.); s.piro@ispro.toscana.it (S.P.); giorgia.stoppa@live.it (G.S.); 3 Piedmont Regional Operations Center of the National Mesothelioma Registry, Unit of Cancer Epidemiology, CPO-Piedmont and University of Turin, 10126 Turin, Italy; enrica.migliore@cpo.it; 4 Tuscan Occupational Cancer Registry and Tuscan Mesothelioma Registry, Occupational and Environmental Epidemiology Branch, Cancer Risk Factors and Lifestyle Epidemiology Unit, Institute for Cancer Research, Prevention and Clinical Network (ISPRO), 50141 Florence, Italy; e.chellini@ispro.toscana.it; 5 Emilia-Romagna Regional Operations Center of the National Mesothelioma Registry, 42122 Reggio Emilia, Italy; romanellia@ausl.re.it; 6 Cancer Registry of the Provinces Catania-Enna-Messina-Siracusa, University of Catania, 95123 Catania, Italy; carlo@carlosciacchitano.it; 7 Lombardy Regional Operations Center of the National Mesothelioma Registry, Occupational Medicine Unit Fondazione IRCCS Ca’ Granda Ospedale Maggiore Policlinico, 20122 Milan, Italy; carolina.mensi@unimi.it; 8 Apulian Regional Operations Center of the National Mesothelioma Registry, School of Medicine, Interdisciplinary Department of Medicine (DIM), Ramazzini Occupational Medicine Section, “Policlinico” University Hospital, University of Bari “A. Moro”, 70124 Bari, Italy; domenica.cavone@uniba.it; 9 Lazio Regional Operations Center of the National Mesothelioma Registry, Department of Epidemiology, Lazio Regional Health Service, 00147 Rome, Italy; e.romeo@deplazio.it; 10 Italian Workers’ Compensation Authority (INAIL), Department of Occupational and Environmental Medicine, Epidemiology and Hygiene, 00143 Rome, Italy; s.massari@inail.it (S.M.); a.marinaccio@inail.it (A.M.); 11 Interdepartmental Centre G. Scansetti for Studies on Asbestos and other Toxic Particulates, University of Turin, 10125 Turin, Italy

**Keywords:** occupational cancer, record linkage, asbestos, mesothelioma, lung cancer, ovarian cancer, larynx cancer, gastrointestinal cancer

## Abstract

The identification and monitoring of occupational cancer is an important aspect of occupational health protection. The Italian law on the protection of workers (D. Leg. 81/2008) includes different cancer monitoring systems for high and low etiologic fraction tumors. Record linkage between cancer registries and administrative data is a convenient procedure for occupational cancer monitoring. We aim to: (i) Create a list of industries with asbestos exposure and (ii) identify cancer cases who worked in these industries. The Italian National Mesothelioma Registry (ReNaM) includes information on occupational asbestos exposure of malignant mesothelioma (MM) cases. We developed using data from seven Italian regions a methodology for listing the industries with potential exposure to asbestos linking ReNaM to Italian National Social Security Institute (INPS) data. The methodology is iterative and adjusts for imprecision and inaccuracy in reporting firm names at interview. The list of asbestos exposing firms was applied to the list of cancer cases (all types associated or possibly associated with asbestos according to International Agency for Research on Cancer (IARC) monograph 100C) in two Italian regions for the indication of possible asbestos exposure. Eighteen percent of the cancer cases showed at least one work period in firms potentially exposing to asbestos, 48% of which in regions different from where the cases lived at diagnosis. The methodology offers support for the preliminary screening of asbestos exposing firms in the occupational history of cancer cases.

## 1. Introduction

The Italian law on the protection of workers (D.Leg. 81/2008) includes a system for the registration of occupational cancers based on three activities: The National Mesothelioma Registry (ReNaM) [1], the Sinonasal Cancer National Registry—ReNaTuns [2], and a system for the identification of clusters of cancer of occupational origin (Occupational Cancer Monitoring—OCM). ReNaM and ReNaTuns follow the same methodology: In each region, a dedicated operational center identifies the cases in hospitals, interviews them on occupational and environmental exposure, and sends the data to a national center for statistical analyses and reporting.

The methodology for OCM has to be different because OCM targets all cancer types with existing evidence of occupational etiology. It would not be possible to collect individual information on occupational exposure interviewing all subjects: In Italy each year, 371,000 new cases of malignant neoplasm are diagnosed, of which 39,000 are lung cancer [3]. Therefore, a different operational model was devised for OCM according to the research conducted by Crosignani et al. [4,5] for the identification of work-related cancer cases. In extreme synthesis, cancer cases and population controls are linked to the archive of pension contributions held by the Italian National Social Security Institute (INPS). The data for each year include the employing company and its industrial sector but does not provide information on actual exposures. Crosignani et al. developed the methodology to use this information in epidemiological analyses to measure the risk by industry and industrial sector, with the case-control study design [4,5]. The record linkage model has the advantage of being applicable on a large scale with very limited resources but has several limits. In particular, the information provided by the record linkage is limited to the identification of the firms where the index subject worked, with no exposure information. Actual exposures have to be estimated using external information, such as JEMs or ‘ad hoc’ investigations carried on by the Occupational Health Departments of the Local Health Authorities.

In the OCM, cancers caused by asbestos exposure are of special interest, given the large use of asbestos materials in Italy, the extent of exposure, and the possibility of compensation for asbestos-related diseases [6]. According to International Agency for Research on Cancer (IARC), cancers caused by asbestos exposure include cancers of the lung, larynx, ovary, and possibly of the pharynx, stomach, and colon, as well as mesothelioma [7]. It is extremely important, therefore, to devise a method for preliminary estimation of asbestos exposure applicable in the OCM. The ReNaM data provide a rich database on industries with evidence of asbestos exposure that could be useful for this purpose. Unfortunately, the first attempts in the use of such information showed that the information in the ReNaM database could not be used directly as an additional input to the linkage process because of the lack of standardization of information reported at interview by MM cases.

The present project aims to improve the evaluation of asbestos exposure in the context of the surveillance and discovery of occupational cancers, using the information on asbestos exposure collected in the Italian MM registry—ReNaM and applying it to the evaluation of asbestos exposure of other cancer cases. In this report, we present the design and implementation of the methodological effort aimed at (i) the identification of the firms with possible asbestos exposure and (ii) the application of this list of firms to the cancer cases identified in the OCM monitoring.

## 2. Materials and Methods

**Figure 1:**
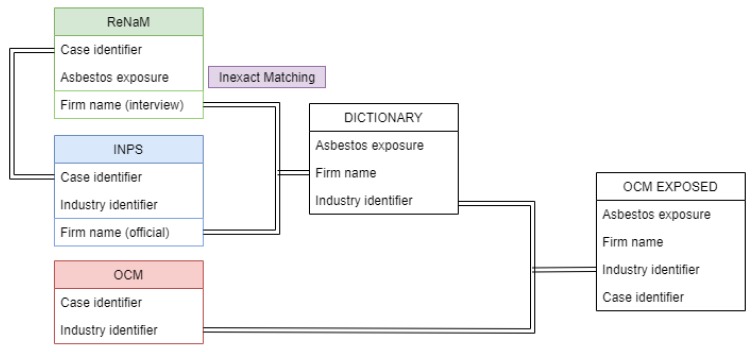
Graphical description of the different record linkage activities. ReNaM—The National Mesothelioma Registry; INPS—Italian National Social Security Institute; OCM—Occupational Cancer Monitoring.

The project was conducted following four steps: (1) MM cases included in the ReNaM database were linked to the INPS database, in order to capture the corresponding firm names and identifiers. (2) The firm names reported for each case in the ReNaM’s interview (reported with possible imprecisions at the interview of MM cases) were associated with the official firm names recorded in the INPS database. (3) Firms with likely exposure to asbestos according to the ReNaM evaluation were listed, identified by INPS firm names and codes, and asbestos exposure rating. (4) The list (thus called ‘dictionary’ in the following) was applied to the occupational histories of the cancer cases from the OCM project for the indication of the firms with possible asbestos exposure. We must remember for understanding step 4 that occupational history for the cancer cases from the OCM project was limited to the information from the INPS database. Figure 1 summarizes the flow of different record linkage activities.

### 2.1. Step 1

The required databases were: The database of the MM cases (ReNaM) and the database of the pension contributions (INPS).

The ReNaM database for this step provided the names and dates of birth of recorded cases.

The INPS database included all pension contributions from private firms from 1974 on. The database included, for each subject and pension contribution, the official registration name and code of the firm. It must be remembered that contributions from the public sector, agriculture, military forces, and self-employed workers were not included in the INPS database.

The linkage was conducted nominally using the MM cases’ names and dates of birth that were linked to the INPS database. In order to reduce the linkage cost, the list of subjects submitted to the linkage was limited to the MM cases with complete demographic data and with evidence of work periods with asbestos exposure according to ReNaM evaluation.

The output from the record linkage in step 1 was the list of firms where the MM cases had worked, according to INPS. It included all firms where a MM case had worked and paid pension contributions, with dates of entry and dismissal. The output record included the official name of the firms and also the address, the Value Added Tax (VAT) code, the economic activity code (coded using both ATECO 91 and ATECO 81), and the job duties of the index subject in broad categories (blue or white-collar).

### 2.2. Step 2

The data entered in step 2 were the output from step 1 and the records of the job history of MM cases from ReNaM.

The methodology of ReNaM for asbestos exposure assessment was described in detail elsewhere [1,8] and was only summarized here for the comprehension of this step of the activity. The ReNaM database included information reported in open words during the interview of MM cases, including the names of the firms where they worked. As always happens in interviews, there were imprecisions in the wording of firm names, often reported differently from their official names. ReNaM occupational hygienists’ rated the level of asbestos exposure for each firm from the job descriptions collected at interview, but kept the firm names as they were reported, with no standardization.

For the purpose of the project, we had to transfer the information on asbestos exposure rated by ReNaM to the official firm names as in the INPS database. The process consisted in the link of the ReNaM database to the output from step 1, by firm names. The linkage cannot be deterministic, due to the different collection of firm identifiers in the ReNaM (interview of MM cases) and in the INPS (official administrative registration) databases. A complex methodology had to be devised and applied to maximize the number of records matched. Our main challenge was the application of approximate string matching or fuzzy name matching to find for each firm name from ReNaM, the corresponding name from the INPS list.

To perform this, we first evaluated and corrected the data, implementing a recursive and iterative pre-processing procedure (text mining and text cleaning [9]). Second, we linked the databases using different approaches and, third, we sent the results to the different ReNaM regional centers for a check by local experts. This last step was used to evaluate and supervise the performance of our algorithm, examining the linked and non-linked data. We also checked a random sample of records to estimate the percentage of correct matches. The procedure was repeated after receiving the amendments by the regions. Eventually, we prepared the final list of asbestos using firms with their official name and INPS registration code.

**Figure 2:**
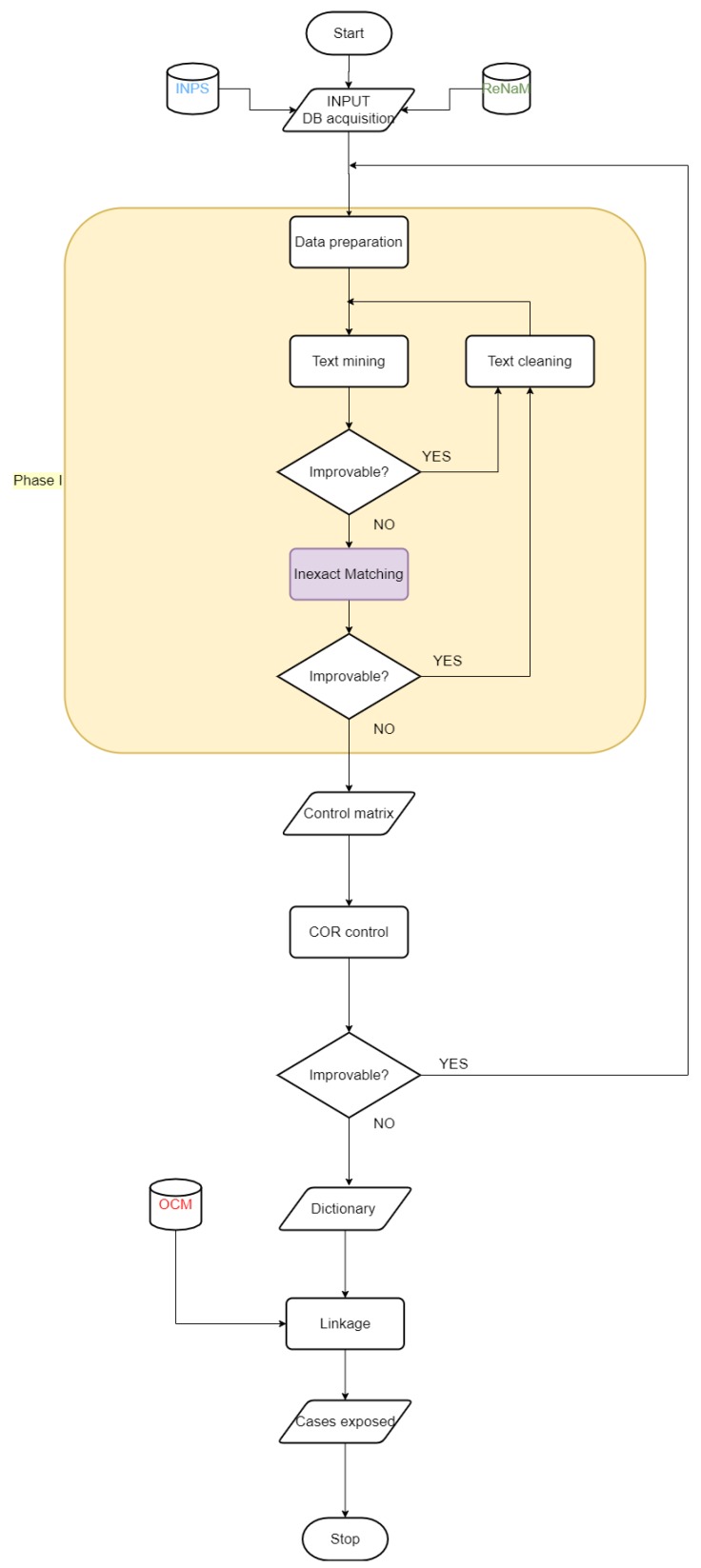
Flowchart of the record linkage process.

The procedure was implemented separately for each region, and the partial-results obtained for each unit were used to increase the capability and competence of the algorithm. The flowchart in Figure 2 shows the procedure, and further details are provided in the next Section 2.2.1 and Section 2.2.2.

#### 2.2.1. Step 2: Text Mining and Text Cleaning

Text mining helped us to understand and discover patterns and anomalies of the firms’ names. The strings with the firms’ names were split into single words in a process called “tokenization”; analyses based on word similarity, term frequency, and word differences were performed with appropriate indices [9]. The validity of the Zipf [10] and Heap laws [11], measures commonly used in quantitative linguistics, was evaluated by studying the relation between the rank of a word and its frequency and between the number of different words and the total number of used words. Terms that tend to co-occur together were also observed using the bi-grams that help to explore pairs of adjacent words [9].

Text cleaning was performed using the information provided by the text mining. Preprocessing of text data such as removing delimiters, numbers, and converting text to upper case was performed. A list of “stop words”, common terms that were not specific or discriminatory, was defined, and they were deleted from the strings. Data were corrected according to sound-like operators [12,13] that studied the inflections, synonyms, and stemming that were the modifications of a word to express different grammatical categories such as tense, case, voice, aspect, person, number, gender, and mood.

After several loop iterations, the 2 datasets were ready for the ‘inexact matching’ linkage procedure.

#### 2.2.2. Step 2: Inexact Matching Linkage

The record linkage between the firm names, as reported at the ReNaM and INPS databases, was used to add the ReNaM evaluation of asbestos exposure to the INPS information.

The linkage algorithm was designed choosing the options that maximized the number of records linked within the regions. The linkage used the single words (tokens) of the firm’s name and not the whole string: For this reason, we used the term “inexact matching”. Before the linkage, records reporting employment in some categories, such as the agricultural sector, the military, and firemen, were excluded from the ReNaM database because these categories were not included in the INPS database (see before for details).

First, we performed an inexact matching within-subject: We considered a correct match when the two datasets shared the cases code and at least one significant word of the firm name.

Second, a full join was performed using the records unmatched in the previous step: All the remaining rows from ReNaM were combined with all records received from INPS. The procedure was time-consuming and, to apply to our big databases, we split the tables into subgroups of 10 rows, selecting only the records that shared at least one word. The remaining records were used and re-controlled in the following step. We decided to consider a successful match when 2 words of the firm name were equal. The choice to use 2 words as a threshold was related to empiric criteria: 1 was too little (excess of sensitivity), and 3 was too big (excess of specificity). In some cases, after the preprocessing phase, the strings were reduced, and they were composed of only one term. In this case, a match was considered successful when the single word present in ReNaM was found in an INPS record.

Third, we searched into the remaining not linked records, the known companies that could be registered with more than one name or with a noun accompanied by other terms. Examples were Enel or Pirelli, big firms with a large number of departments and subgroups. To avoid the possible loss of the records, we forced a list of the company’s names known ‘a priori’.

**Figure 3:**
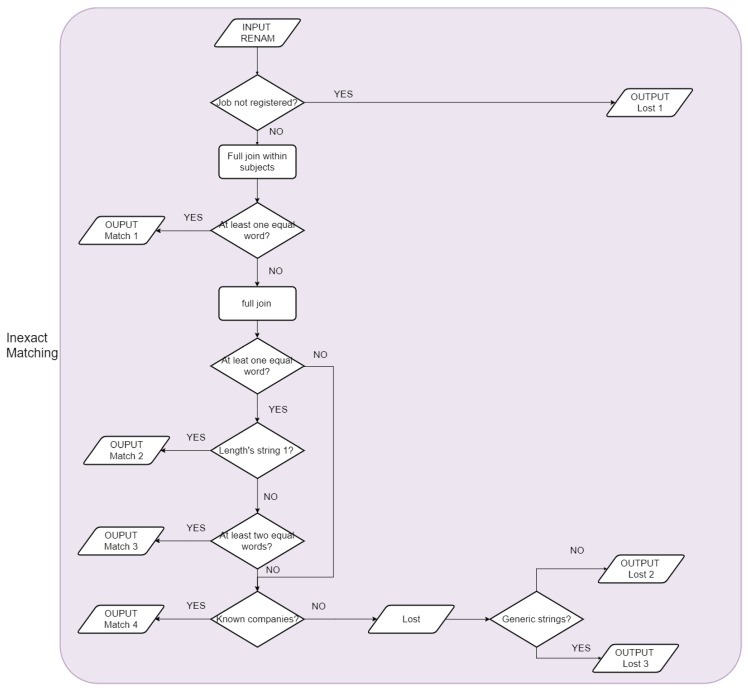
Record linkage flowchart: ‘inexact matching’ steps.

The ReNaM remaining unmatched records were reorganized into 3 groups: ‘a priori’, ‘generic terms’, and ‘not generic term’ to be controlled by the regional experts. The process was repeated after their input. Figure 3 presents the record linkage flowchart, expanding the flowchart in Figure 2.

### 2.3. Step 3

The result of the merge procedures was a general table (“dictionary”), including all firms with evidence of asbestos exposure. Each firm record included: The official name of the firm as from INPS, its official administrative registration number, the rating of exposure to asbestos (certain, probable, possible) according to ReNaM experts and the firm name as reported at the ReNaM interviews. Duplicates were reduced to a single record, keeping the higher asbestos exposure rating.

The results of the merge procedure were validated by regional experts in each regional unit of the ReNaM.

The firms’ addresses were geocoded [14,15], and the latitude and longitude coordinates were used to perform spatial analysis.

### 2.4. Step 4

The application of the general table (“dictionary”) of asbestos exposing firms to the working histories of cancer cases from the OCM project was conducted for the 2 Italian regions (Lazio and Sicily), according to the OCM data available at the time of the research. All cancer types associated or possibly associated with asbestos according to IARC [7], were included. The information included for each case were: The diagnosis, the date of diagnosis, and the occupational history as provided by the INPS (firm names and codes). The output was the cases with at least a firm registration number present in the dictionary, i.e., the subjects who worked in a probably asbestos-exposed industry.

Steps 1 to 3 were conducted using the information on MM cases incident in 1993–2012 in 7 Italian regions separately (Piedmont, Lombardy, Tuscany, Emilia-Romagna, Lazio, Apulia, and Sicily). Step 4 involved only 2 Italian regions corresponding to the extension of the OCM project at the time of our analysis: We used the Lazio’s hospital discharge in the period 2008–2015 and cancer cases in Eastern Sicily in the period 2011–2014.

All analyses were performed using SAS 9.3 (SAS Institute Inc., Cary, NC, USA), R version 3.4.1 (R Core Team, Vienna, Austria), STATA 11 (StataCorp LLC, College Station, TX, USA).

### 2.5. Ethics

This project did not involve any contact with subjects, and the use of personal information was authorized by the Italian law on the protection of workers (D.Leg. 81/2008), therefore, approval from the Institutional Ethics Committee was not required.

## 3. Results

For clarity, the results were presented separately for the different steps described in the Material and Methods.

### 3.1. Step 1

The ReNaM included MM cases diagnosed between 1993 and 2012 in the seven Italian regions involved in the project [1]. In our project, we included 6057 cases: The remaining cases were excluded ‘a priori’ because they did not report complete identification data, or names of firms, or lacked any evidence of occupational asbestos exposure. Of them, 4134 (68.25%) were successfully linked with INPS (see MM step 1). Table 1 presents the results by region. The table presents the number of records, corresponding for ReNaM to the number of employments reported at the interview and for INPS to the number of different firms recorded in the database for the linked subjects. The number of records was higher than the cases because a subject could have worked in more than one firm. The regions with the higher number of cases where Lombardy (2276) and Piedmont (1104), while for Sicily and Lazio fewer subjects with MM were observed: 256 and 289, respectively, corresponding to the different incidence of MM and prevalence of occupation in industries contributing to INPS.

Table 2 describes the occupational records of the linked MM cases according to the level of occupational asbestos exposure rated by ReNaM, expanding the corresponding column of Table 1.

### 3.2. Step 2

Data entered in step 2 were the records of the job history of MM cases.

The results of the linkage procedure conducted in step 2 to analyze the lists of firms (details in Materials and Methods) are presented in Table 3. ReNaM records reporting agricultural, military, and firemen sectors were ‘a priori’ excluded because these occupational sectors were not in the INPS database and, therefore, linkage was impossible. These excluded records ranged from 3% to 25% of ReNaM records, depending on the region considered. The remaining records were analyzed, and we considered a successful match when the records shared (1) at least one word into the same subjects, (2) at least two words, (3) one word in length’s string equal to one. Then, in (4), we selected the companies in the ‘a priori list’ of known companies.

The total matched records were 5327 (41.61%), but a huge variation was observed between regions. Better performance was observed in Piedmont (52.37%) followed by Emilia Romagna (46.23%), Sicily (45.91%), and Lombardy (45.30%) while the percentage of records linked was lower in Tuscany (29.99%), Lazio (31.39%), and Apulia (35.23%). If we do not consider the ‘a priori’ exclusions in the denominator, the percentage of successful matches was higher, particularly for Apulia (47.26%), Emilia Romagna (52.67%), and Lazio (35.99%) (results not tabulated).

### 3.3. Step 3

The numbers of different firms obtained from the linkage procedure are shown in Table 4, by region and level of asbestos exposure. As explained in the Material and Methods section, whenever different ratings were present for the same firm, we considered the highest rating, and when the same firm was shown in different regions, we considered it only once. Regions with more MM cases contributed with more firms, and particularly, Lombardy contributed for 40% (1080 firms) of the national total. The total number of unique firms identified was 2606: 1826 (70.07%) with certain, 222 (8.52%) with probable, and 558 (21.41%) with possible asbestos exposure. The list of official INPS firm names and codes formed the thus called ‘dictionary,’ to be used for the evaluation of asbestos exposure in the next step of the process.

**Figure 4:**
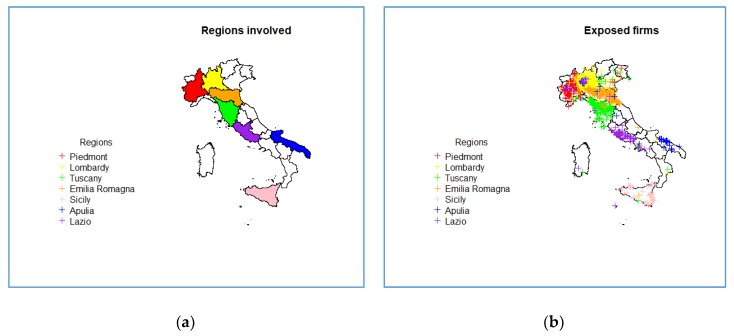
(**a**) Italian map of regions involved in the study. (**b**) Italian map with the spatial distribution of exposed firms.

One of the advantages of this process is the provision of a list of ‘asbestos exposing firms’ that is not limited to the experience of regional experts. In Figure 4, we reported the two Italian maps with the regional boundaries. In the left panel (Figure 4a), we highlighted the regions involved, while in the right (Figure 4b), the spatial distribution of exposed firms, separately the region of origin of the MM cases, was reported.

### 3.4. Step 4

The cases of cancer included in this test on the preliminary identification of asbestos exposing firms in the OCM system were 35,010, 9848 (28.13%) from Sicily and 25,162 (71.87%) from Lazio, selected from the local population cancer registry (Sicily) or the hospital discharge (Lazio) databases. Table 5 reports the distribution by tumor type. In the case of multiple malignancies in the same subject, only the first malignant neoplasm was selected.

We applied the ‘dictionary’ obtained from Step 3 to the list of INPS records for these cases. The cases who had worked in firms selected for asbestos exposure were 1454 and 5092, from Sicily and Lazio, respectively. It indicated that, excluding pleural malignancies, the 18.5% (6400) of the cancer cases extracted for the OCM system could be related to occupational asbestos exposure (Table 6).

About 50% of the records matched were identified using the dictionary information obtained from other regions: 1608 (51.59%) for Sicily and 3463 (46.56%) for Lazio (Table 7).

## 4. Discussion

Exposure to asbestos fibers causes a range of different malignancies, and it is, therefore, highly relevant for any system of detection of occupational cancer. Population health surveillance related to asbestos diseases have different objectives, including: (i) The case detection and notification; (ii) the estimation of reliable epidemiological figures with the identification of outbreaks of disease; (iii) the identification of possible sources of contamination still in place and the prevention of asbestos exposure; (iv) the planning and implementation of public health policies; (v) the support of the effectiveness of insurance systems and the evaluation of the compensation, (vi) the dissemination of results and documentation of the impact of an intervention [16,17].

The present project aims at contributing to Italian surveillance on occupational cancer with the identification of asbestos exposing firms and the identification of cancer cases who had worked in asbestos-exposed firms.

The exploration of the asbestos-related etiology of cases requires enormous resources, given the relatively low attributable fraction and the high numbers. The objectives were achieved with record linkage connecting information from ReNaM and from INPS through a complex methodology based on the processing of textual information and probabilistic matching. One of the results was the preparation of a list of firms with possible exposure to asbestos in the past, ready for use as a screening tool in new research projects. This list was applied to the first available data from OCM, the occupational cancer monitoring system that is under preparation.

Research heavily uses record linkage, a procedure that is becoming more and more common in statistical and academic research. Linking records makes it possible to combine data from different sources to answer research questions that are very difficult to answer using data from just one source [18,19,20]. In many situations, record linkage is an efficient way to collect data and it can reduce the inconvenience of asking sensitive questions [21,22]. Record linkage is commonly used in Nordic countries to evaluate cancer risk, even in very complex study designs [23].

The use of surveillance systems of data collected by institutional subjects is both an advantage and a disadvantage. From one side, it reduces the duration and cost of the study and allows a standardized classification of the disease, of the occupational histories, and of exposure estimation. On the other hand, the databases that were not created for scientific or research purposes often lack critical information. In particular, the INPS database does not include enough details on occupational exposure, which is the reason for our research.

The differences in the databases limit the overlap of the two systems we used. The ReNaM database was used only for the cases with certain, probable, and possible occupational exposure as evaluated from the interview data. The INPS database excluded some important occupational categories (agriculture, public employment, armed forces) and self-employees, mainly artisans and shopkeepers. Moreover, it includes only contributions from 1974 onwards [4].

Data pre-processing was necessary to optimize the information in the administrative databases before the linkage. The application of text mining and text cleaning techniques have been adopted in various successful biomedical applications in recent years. We applied them to extract meaningful information from the firm names written in open words. A great effort was done to prepare the list of “stop words” and to correct and simplify the terms. Different procedures and algorithms were previously available and were routines implemented in the main statistical software, but the majority of them were based on the English language. Therefore, we had to develop algorithms for the Italian setting. These algorithms are available for use in other contexts or with other languages.

The main challenge in our context was the definition of the linkage key between the names of firms reported with different accuracy in the two databases: More loosely in the ReNaM interviews and more strictly in the INPS. We discarded the deterministic linkage methods because they rely on the presence of common uniquely identifying attributes across all sources. We preferred the probabilistic approach using non-unique attributes and calculating similarity indexes for pairwise comparisons. The quality of linkage procedures is difficult to determine, and this constitutes a major issue in record linkage. In the analysis of the process and results of record linkage, both missed links (false negatives) and spurious links (false positives) must be addressed. Missed links are ‘false negatives’, corresponding to the absence of ‘signals’ of asbestos exposure: The corresponding firms will not be included in the list of asbestos exposing firms. As the process is iterative, this error is expected to be reduced with the future extension of the process. Firms indicated by the link but without exposure are the ‘false positive’ signals that can be corrected by the local expert’s revision. Another limitation regards the very large firms with different plants that are not recognized separately by the linkage. For all these reasons, our procedure must be considered only a screening tool with information that must be validated by the local experts evaluating the actual occupational history of the cases [24].

The algorithm produced a list of firms with potential asbestos exposure that was linked to the occupational histories of cancer cases provided by the OCM system, for the detection of their association to asbestos exposure. Results showed that 18.5% of the firms where the cancer cases other than MM worked had an indication of possible exposure to asbestos.

We provided information extended to the entire country and, therefore, additional to the knowledge of local experts: About 50% of the links referred to firms in regions different from the region of the residence of the case. The graphical distribution of the firm addresses is an effective tool to explore this aspect, which will also be useful for the improvement of interview information in ReNaM.

The process is cost-saving and can be repeated whenever new information is gathered. We are not aware of similar activities using the information from mesothelioma registries for the identification of asbestos exposure of other tumor types. The first extension that can be planned is the nationwide extension of the project, now limited to seven regions, and the evaluation of occupational asbestos exposure for the national OCM system.

## 5. Conclusions

The record linkage from Mesothelioma registries and administrative databases is a valuable tool for the identification of asbestos exposure. Our research provided a useful indicator easily applicable to surveillance studies regarding all cancer types with interest in the evaluation of exposure to asbestos.

## Figures and Tables

**Table 1:** The number of malignant mesothelioma (MM) cases included in The Italian National Mesothelioma Registry (ReNaM) and the number of subjects linked (*n*, %) to the Social Security Institute (INPS) database, and the corresponding number of occupational records, by region.

Region	ReNaM Cases	INPS Linked	ReNaM Records	INPS Records
Piedmont	1104	742 (67.21%)	2490	1885
Lombardy	2276	1595 (70.08%)	3965	4083
Emilia-Romagna	891	634 (71.16%)	1367	1906
Tuscany	906	568 (62.69%)	3465	1600
Lazio	289	189 (65.40%)	634	613
Apulia	335	208 (62.09%)	440	642
Sicily	256	198 (77.34%)	440	873
**Total**	**6057**	**4134 (68.25%)**	**12,801**	**11,602**

**Table 2:** Distribution of occupational asbestos exposure levels by region.

Region		Occupational Asbestos Exposure					
	Total	Certain		Probable		Possible	
	*n*	*n*	%	*n*	%	*n*	%
Piedmont	2490	1480	59.44	208	8.35	802	32.21
Lombardy	3965	2797	70.54	274	6.91	894	22.55
Emilia-Romagna	1367	883	64.59	239	17.48	245	17.92
Tuscany	3465	2464	71.11	388	11.2	613	17.69
Lazio	634	152	23.97	176	27.76	306	48.26
Apulia	440	204	46.36	96	21.82	140	31.82
Sicily	440	177	40.23	119	27.05	144	32.73
**Total**	**12,801**	**8157**	**63.72**	**1500**	**11.72**	**3144**	**24.56**

**Table 3:** The number of ReNaM records and results of the record linkage, by region. The results of linkage procedures were reported to distinguish between excluded (‘a priori’), non-matched, and matched: (1) Within subjects with more than one equal word in firm’s name, (2) two equal words, (3) one equal word in a string of length equal to one, (4) known firms.

Region	ReNaM *n*	Excluded *n* (%)	Matched (By Matching Step)				Matched Total *n* (%)	Non-Matched *n* (%)
			1	2	3	4		
			*n* (%)	*n* (%)	*n* (%)	*n* (%)		
Piedmont	2490	185 (7.43)	861 (34.58)	156 (6.27)	168 (6.75)	119 (4.78)	1304 (52.37)	1001 (40.2)
Lombardy	3965	128 (3.23)	1324 (33.39)	271 (6.83)	106 (2.67)	95 (2.4)	1796 (45.3)	2041 (51.48)
Emilia-Romagna	1367	167 (12.22)	452 (33.07)	71 (5.19)	106 (7.75)	3 (0.22)	632 (46.23)	568 (41.55)
Tuscany	3465	364 (10.51)	755 (21.79)	117 (3.38)	146 (4.21)	21 (0.61)	1039 (29.99)	2062 (59.51)
Lazio	634	81 (12.78)	161 (25.39)	12 (1.89)	2 (0.32)	24 (3.79)	199 (31.39)	354 (55.84)
Apulia	440	112 (25.45)	117 (26.59)	7 (1.59)	22 (5)	9 (2.05)	155 (35.23)	173 (39.32)
Sicily	440	32 (7.27)	163 (37.05)	23 (5.23)	8 (1.82)	8 (1.82)	202 (45.91)	206 (46.82)
**Total**	**12,801**	**1069 (8.35)**	**3833 (29.94)**	**657 (5.13)**	**558 (4.36)**	**279 (2.18)**	**5327 (41.61)**	**6405 (50.04)**

**Table 4:** The number of firms listed for possible asbestos exposure after the linkage, and distribution of occupational exposure, by region. The total number is smaller than the sum of the number of firms in each region because the firm’s names observed in two or more regions were counted only once.

Regions	Matched Records *n*	Firms *n*	Asbestos Exposure Rating of the Firm		
			Certain	Probable	Possible
			*n* (%)	*n* (%)	*n* (%)
Piedmont	1304	429	267 (62.24)	25 (5.83)	137 (31.93)
Lombardy	1796	1080	872 (80.74)	41 (3.80)	167 (15.46)
Emilia-Romagna	632	370	259 (70.00)	38 (10.27)	73 (19.73)
Tuscany	1039	537	420 (78.21)	45 (8.38)	72 (13.41)
Lazio	199	129	33 (25.58)	50 (38.76)	46 (35.66)
Apulia	155	99	50 (50.51)	10 (10.10)	39 (39.39)
Sicily	202	142	57 (40.14)	34 (23.94)	51 (35.92)
**Total**	**5208**	**2606**	**1826 (70.07)**	**222 (8.52)**	**558 (21.41)**

**Table 5:** Distribution of malignant neoplasm in occupational cancer monitoring (OCM) data for Lazio and Sicily.

Malignant Neoplasm	Region			
	Lazio (*n* = 25,162)		Sicily (*n* = 9848)	
	*n* Cases	%	*n* Cases	%
Colorectal	9373	37.25	3812	38.71
Pharynx	605	2.4	137	1.39
Larynx	1288	5.12	573	5.82
Ovary	1490	5.92	464	4.71
Pleura	221	0.88	196	1.99
Lung	9590	38.11	3650	37.06
Nasopharynx	196	0.78	140	1.42
Stomach	2399	9.53	876	8.9

**Table 6:** Distribution of malignant neoplasm in OCM data (all and matched cases) for Lazio and Sicily.

Malignant Neoplasm	Region			
	Lazio Cases		Sicily Cases	
	*n*	Matched	*n*	Matched
Colorectal	9373	2020	3812	579
Pharynx	605	113	137	17
Larynx	1288	248	573	81
Ovary	1490	169	464	28
Pleura	221	83	196	53
Lung	9590	1953	3650	561
Nasopharynx	196	35	140	12
Stomach	2399	471	876	123
**Total**	**25,162**	**5092**	**9848**	**1454**

**Table 7:** The number of OCM records and cases for Sicily and Lazio. The number of linked records was divided into the records identified using the information from the region (in region) or the dictionary records from the other regions (out region).

Region	OCM		Matched			
	Records *n*	Cases *n*	Records			Cases *n*
			All *n*	In Region *n* (%)	Out Region *n* (%)	
Lazio	89,274	25,162	7437	3974 (53.44)	3463 (46.56)	5092
Sicily	47,602	9848	3117	1509 (48.41)	1608 (51.59)	1454
**Total**	**136,876**	**35,010**	**10,554**	**5483 (51.95)**	**5071 (48.05)**	**6546**
